# Longitudinal radiomics of liver metastases during magnetic resonance-guided radiotherapy

**DOI:** 10.1016/j.phro.2026.101094

**Published:** 2026-09-26

**Authors:** Alina Paunoiu, Maksym Fritsak, Hubert S. Gabryś, Sebastian M. Christ, Matthias Guckenberger, Stephanie Tanadini-Lang, Riccardo Dal Bello

**Affiliations:** a Department of Radiation Oncology, University Hospital of Zürich and University of Zürich, Zürich, Switzerland; b Faculty of Medicine, University of Zürich, Zürich, Switzerland

**Keywords:** Liver, MRgRT, Radiomics, Longitudinal

## Abstract

**Background and purpose:**

Magnetic resonance-guided radiotherapy (MRgRT) offers high-quality soft tissue contrast and treatment monitoring, supporting longitudinal radiomics analysis. We investigated treatment-related radiomic changes during stereotactic body radiotherapy (SBRT) for liver metastases and healthy liver.

**Materials and methods:**

Features were extracted from the gross tumour volume (GTV) and five 2-cm liver rings around the planning target volume (PTV) in 34 patients treated with five-fraction MRgSBRT. Features showing changes below baseline variability or high correlation (Spearman's |r| > 0.9) were excluded, with pre-treatment values averaged into a robust baseline. Linear regression slopes informed uniform manifold approximation and projection (UMAP) followed by hierarchical density-based spatial clustering of applications with noise (HDBSCAN) for patient stratification, with false discovery rate-corrected Kruskal–Wallis testing identifying representative features. Correlations with GTV *volume* and mean cumulative liver-ring dose were evaluated.

**Results:**

Of 160 GTV features, 55 uncorrelated features remained. Three patient groups (*n* = 4, 10, and 20) with distinct longitudinal patterns were identified. *Intensity-based energy* showed significant differences across patient groups (adjusted *p* = 0.001), weakly correlated (|r| < 0.7) with *volume* in the two larger groups. In the innermost liver ring, 49 uncorrelated features were analysed. Two patient groups (*n* = 19 and 15) were identified, with *intensity-based energy*, *mean intensity*, and *maximum intensity* showing significant group differences, weakly correlated with mean cumulative dose.

**Conclusions:**

This longitudinal MR-based radiomics framework identified GTV and healthy liver patient groups, with distinct treatment-related feature changes. This pipeline could be extended to integrate treatment response assessment in future studies.

## Highlights

• Magnetic resonance-guided radiotherapy enabled longitudinal radiomics monitoring.
• Radiomic features from tumour and healthy liver enabled patient stratification.
• Distinct temporal radiomic patterns were identified across patient groups.
• *Intensity-based energy* differed significantly between patient groups (*p* ≤ 0.001).
• Representative features were largely independent of tumour volume and liver dose.

## Graphical abstract

**Figure:**
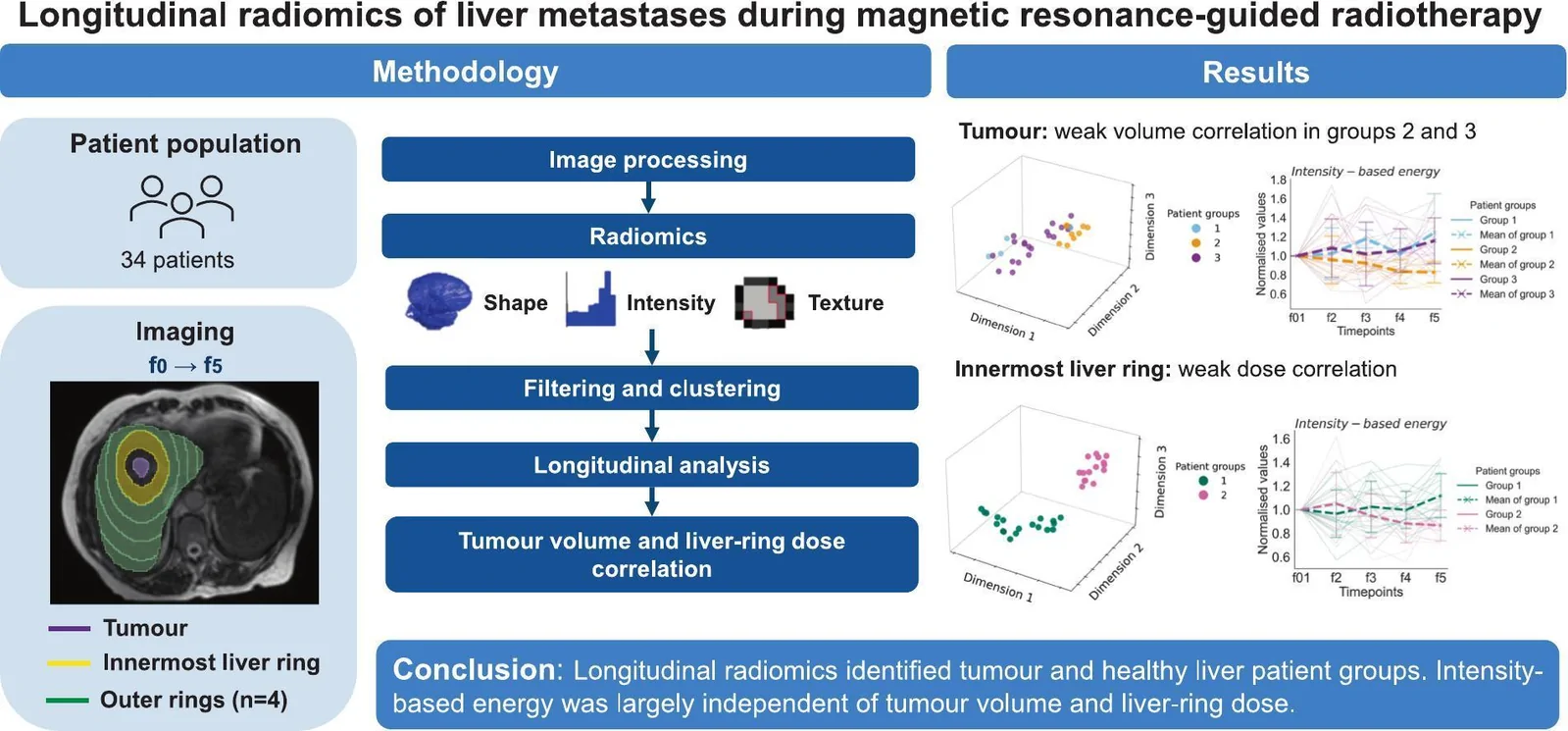

## 1 Introduction

The liver is one of the most frequent sites of metastatic spread from several primary malignancies, particularly those arising from the gastrointestinal (GI) tract, breast, and lung. This is due to the liver's vascular anatomy and metabolic environment [1], [2].

For patients with a small number of liver metastases, classified as oligometastatic disease, surgical resection remains the standard of care when feasible, with reported 5-year overall survival (OS) of up to 50% in colorectal cancer [1]. However, approximately 80% of patients are not amenable to surgery due to either the location of the metastases or poor performance status. For these patients, stereotactic body radiotherapy (SBRT) has emerged as a promising non-invasive alternative [3]. Common fractionation schemes include 45 Gy to 50 Gy delivered in three to five fractions, typically prescribed to the 65% or 80% isodose line [4].

Recent advancements in image guidance have further refined this approach, with the development of magnetic resonance-guided radiotherapy (MRgRT), enabled by the hybrid magnetic resonance linear accelerator (MR-linac) technology [5]. Such systems allow repeated MR image acquisitions, offering high-quality soft tissue contrast and real-time tracking capabilities to monitor changes over the course of treatment [2], [4], [6], [7], which can be leveraged for quantitative medical image analysis (radiomics). Radiomics has emerged as a promising approach for non-invasive cancer characterisation and refers to the extraction of a large number of quantitative features from medical images, such as computed tomography (CT), positron emission tomography (PET), or MRI [1]. These features are broadly divided into morphology, intensity/histogram and texture features [8], [9], [10].

The potential of radiomics to identify prognostic imaging-based biomarkers has been demonstrated in several cancer types, including cancers of the liver, pancreas, rectum, and lung [11], [12], [13], [14], [15], [16], [17], [18], [19]. Boldrini et al. [13] evaluated delta radiomics extracted from the gross tumour volume (GTV) from patients treated with hybrid MRgRT for rectal cancer. They identified two features predictive of clinical complete response (cCR) (*p* = 0.001). Tomaszewski et al. [14] focused on histogram and texture features in pancreatic adenocarcinoma and showed that the ratio of histogram *skewness* between the first and fifth fractions strongly correlated with progression-free survival (PFS) (*p* = 0.005), suggesting its potential as an early response indicator. Jin et al. [15] analysed MRI-based delta-radiomic features to predict local control in liver lesions treated with MR-guided SBRT, reporting two texture features as the most predictive, with an area under the curve (AUC) of 0.72–0.90 depending on the biologically effective dose.

Despite the growing body of evidence, no study to date has characterised per-fraction longitudinal radiomic changes in both the GTV and the surrounding healthy liver tissue during MR-guided SBRT for liver metastases. We hypothesised that such treatment-related feature trajectories might reflect biological changes not captured by gross tumour volume alone and could be used to identify distinct patient groups with differing longitudinal response patterns. Building on this, we also aimed to evaluate whether these temporal patterns are associated with the GTV volume or the mean cumulative radiation dose to the surrounding healthy liver tissue.

## 2 Materials and methods

The workflow described below is summarised schematically in Fig. S1.

### 2.1 Patient cohort

Thirty-four patients with a single liver metastasis were included in the study, treated with MR-guided SBRT at the Department of Radiation Oncology of the University Hospital Zürich between December 2019 and April 2022. MR images were acquired with the 0.35 T ViewRay MRIdian system at the time of pre-treatment simulation (f_0_) and before each SBRT fraction was delivered (f_1_-f_5_) [20].

To account for histological heterogeneity of the primary tumour, we grouped the patients into four broader categories: ‘Breast & Gynecological’, ‘GI & Biliary’, ‘Lung & Head & Neck’, and ‘Other’. These groupings were defined with a clinical expert to ensure interpretability while preserving biological relevance. Patient characteristics are summarised in Table S1. The fractionation scheme was determined by the treating physician and ranged from 5 × 5 Gy to 5 × 9 Gy, all prescribed to the 65% isodose line. The majority of patients received 5 × 9 Gy (17/34), followed by 5 × 8 Gy (10/34), 5 × 7 Gy (5/34), 5 × 6 Gy (1/34), and 5 × 5 Gy (1/34) (Fig. S2). This retrospective analysis was approved by the cantonal ethics committee Zurich (BASEC-Nr. 2018–01794) in accordance with the 1964 Declaration of Helsinki and its later amendments. All patients gave their consent for retrospective data analysis [21].

### 2.2 Imaging and radiomic feature extraction

In total, 204 MR scans, acquired using a True Fast Imaging with Steady State Precession (TRUFI) sequence [5], were analysed. Two scans were acquired at each fraction: the first to check and optimise patient positioning and the second to confirm correct positioning [2]. For our analysis, the first scan was used at each fraction.

All structures were manually delineated in the ViewRay treatment planning system by the treating physician, post-processed in MIM (version 7.1.6, GE HealthCare, Cleveland, USA) and exported for analysis. DICOM images were converted to NIfTI format using our software, *Z*-Rad (version 25.2) [22], [23].

Our analysis focused on the GTV and five three-dimensional concentric ring structures surrounding the planning target volume (PTV), representing the healthy liver tissue. These structures, hereafter rings, were generated using the NumPy, SimpleITK and SciPy packages in Python (version 3.12.2), with a width of 2 cm and cropped at the liver contour. The images were processed using bias field correction and z-score normalisation, also implemented using the SimpleITK and SciPy packages, followed by resampling from 1.5 × 1.5 × 3 mm^3^ to 2 × 2 × 2 mm^3^ isotropic voxels.

Radiomic features were extracted from the GTV and the five liver rings at all timepoints using *Z*-Rad. Image intensities for texture feature calculation were discretised using a fixed bin number of 32 bins. Texture features were computed in three dimensions and averaged across directions. Morphological features were excluded from the analysis of the liver rings, as the ring-shaped regions were artificially defined and do not represent actual liver morphology.

### 2.3 Image analysis

For the GTV-based features, we first established the absolute difference between f_0_ and f_1_ as a clinically relevant baseline, as the images at these two timepoints were acquired prior to treatment delivery. Features exhibiting smaller changes in subsequent fractions than this baseline difference were considered below baseline variability and excluded. To reduce feature redundancy, highly correlated features (Spearman's |r| ≥ 0.9) were removed using a sequential approach, in which each feature was compared against the remaining features and, once retained, any feature correlated with it above this threshold was excluded.

To obtain a robust pre-treatment reference, feature values at f_0_ and f_1_ were averaged into a single timepoint, referred to as f_01_. Temporal feature evolution was characterised using linear regression slopes, computed for each feature and patient.

Based on these slope values, we identified patient groups using uniform manifold approximation and projection (UMAP) followed by hierarchical density-based spatial clustering of applications with noise (HDBSCAN) [24]. The optimal number of clusters was determined using the density-based cluster validation (DBCV) metric. DBCV is a validation metric that quantifies clustering quality by simultaneously assessing within-cluster density connectedness and inter-cluster separation. The score ranges from −1 to 1, with higher values indicating better-defined, separated clusters. The clustering solution with the highest DBCV score was considered optimal and used for the subsequent analyses [25].

Differences in feature values across patient groups were assessed using the Kruskal–Wallis test with false discovery rate (FDR) correction. Adjusted *p* < 0.05 was considered statistically significant. The association between histological categories and the feature-derived patient groups was evaluated using a chi-square test.

To evaluate whether representative features were surrogates for changes in GTV volume or carried additional independent information, we calculated Spearman's |r| between each feature and the GTV *volume* feature across all fractions, per patient. Correlations were categorised as weak (|r| < 0.7) or strong (|r| ≥ 0.7).

For the analysis of the surrounding healthy liver tissue, the same feature processing and clustering pipeline was applied. Correlations were computed between significant features and the mean cumulative dose delivered to each liver ring, also using Spearman's |r|, to assess radiation-induced changes in healthy liver tissue.

In both cases, for each feature, we computed the number of patients per group showing strong (|r| ≥ 0.7) versus weak (|r| < 0.7) correlations with GTV *volume* or mean cumulative dose to the liver rings.

## 3 Results

In total, 160 radiomic features were extracted from the GTV, of which 55 were used after removing highly correlated features and features with treatment-induced changes below baseline variability. Based on the temporal feature slopes, UMAP (3D projection) followed by HDBSCAN clustering identified three patient groups (Fig. 1): group 1 (*n* = 4 patients), group 2 (*n* = 10 patients) and group 3 (*n* = 20 patients). The global DBCV score was 0.58, while the DBCV score for groups 2 and 3 evaluated together was 0.65.

**Fig. 1:**
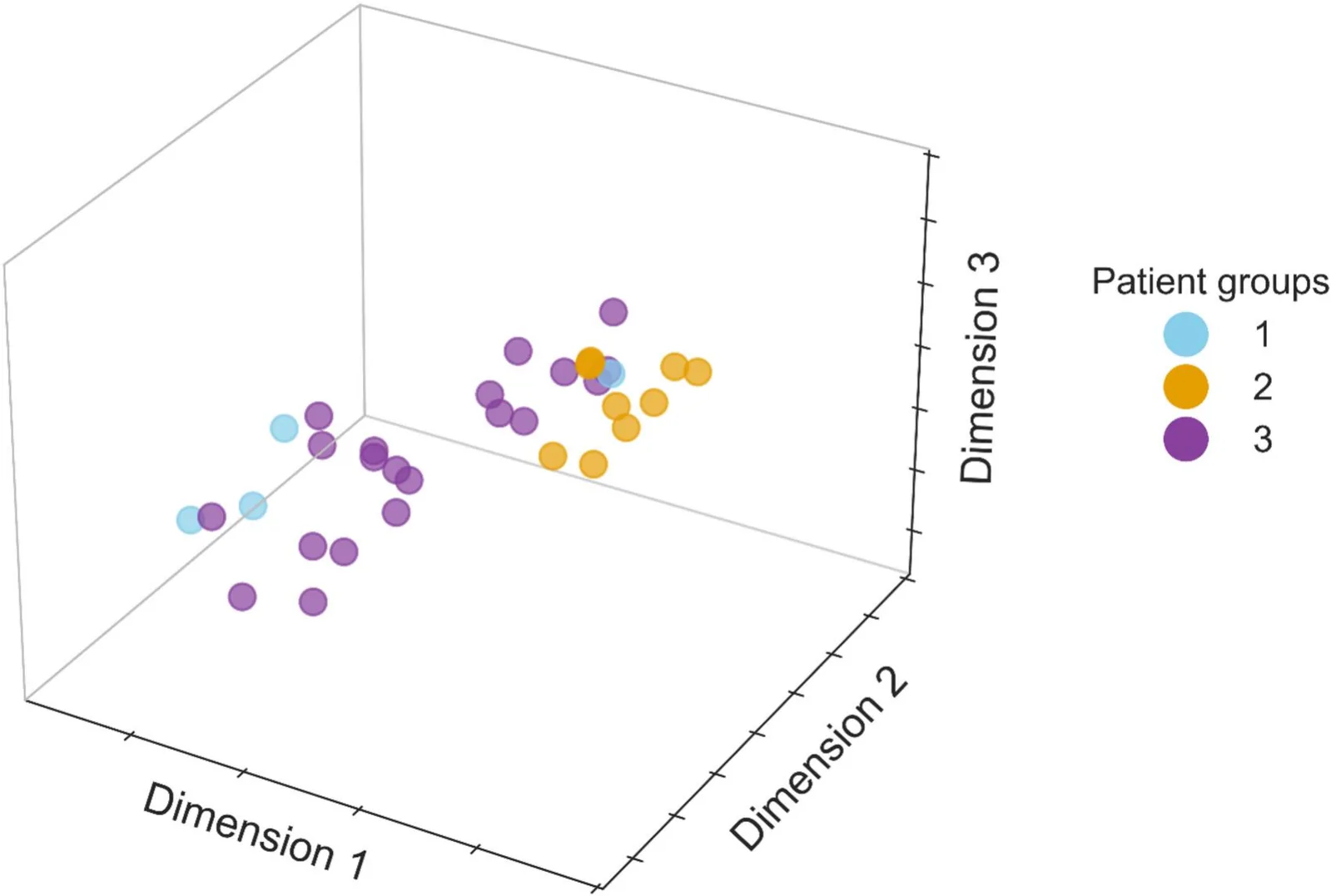
Three-dimensional uniform manifold approximation and projection (UMAP) of gross tumour volume (GTV)-based patient groups. Hierarchical density-based spatial clustering of applications with noise (HDBSCAN) based on temporal GTV feature slopes identified three patient groups: group 1 (blue, *n* = 4), group 2 (yellow, *n* = 10) and group 3 (purple, *n* = 20). (For interpretation of the references to colour in this figure legend, the reader is referred to the web version of this article.)

Among the evaluated GTV features, *intensity-based energy* showed a significant difference in its longitudinal evolution across the three patient groups (adjusted *p* = 0.001). Group-level trajectories normalised to f_01_ are shown in Fig. 2 and individual patient trajectories in Fig. S4A. Group 2 showed a gradual decrease over the course of treatment, whereas groups 1 and 3 demonstrated stable to slightly increasing trends, with separation becoming more pronounced toward later fractions. No significant association was found between the histological categories and the three feature-derived patient groups (*p* = 0.384), with all primary tumour types represented in each group (Fig. S3).

**Fig. 2:**
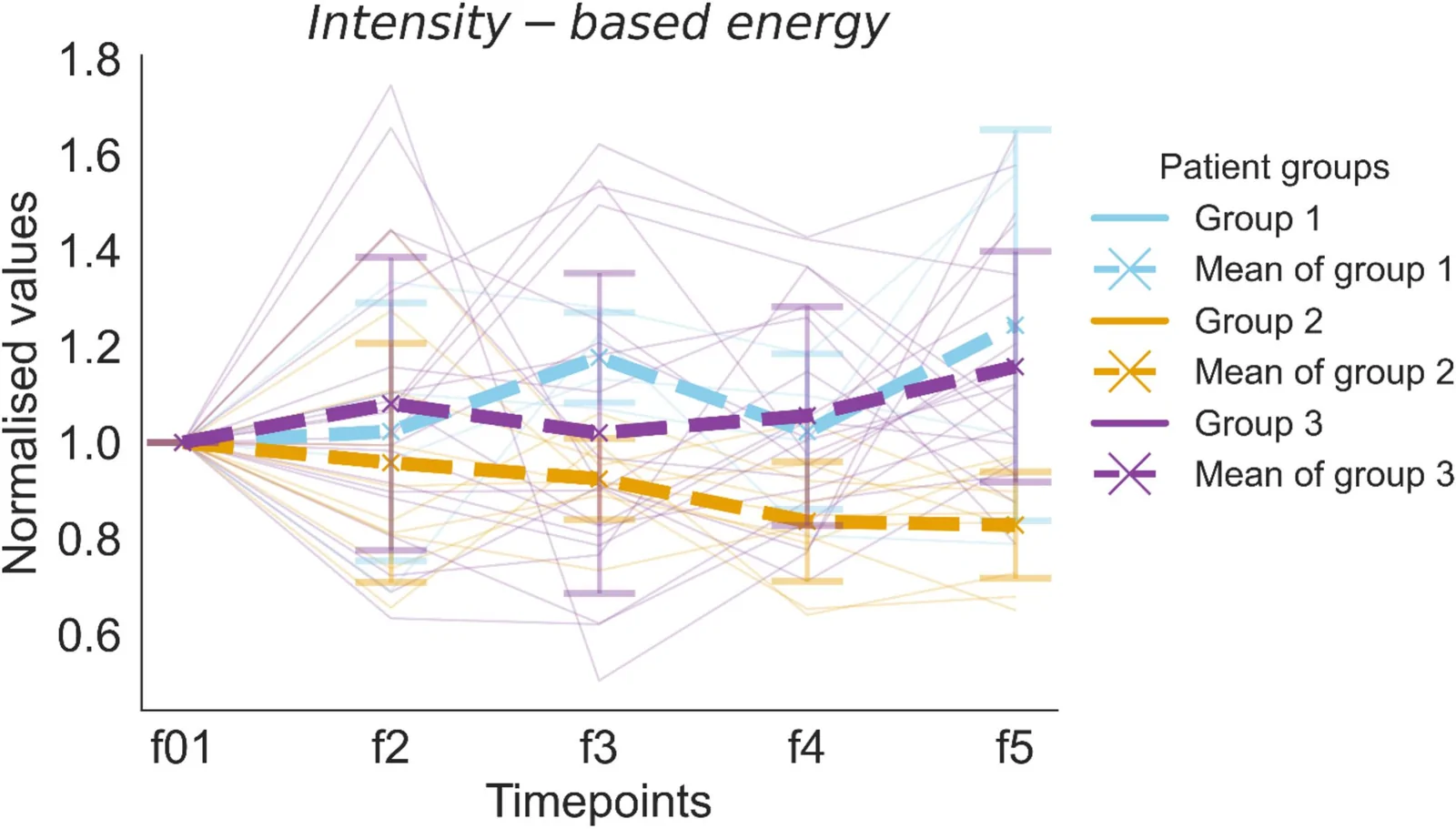
Longitudinal evolution of gross tumour volume (GTV) *intensity-based energy*. Solid lines indicate individual trajectories shown for the three patient groups (group 1: blue, *n* = 4; group 2: yellow, *n* = 10; group 3: purple, *n* = 20). Dashed lines represent group means, and error bars denote the standard deviation within each group. (For interpretation of the references to colour in this figure legend, the reader is referred to the web version of this article.)

The same feature exhibited a higher number of strong correlations with the GTV *volume* (|r| ≥ 0.7) in patient group 1, while groups 2 and 3 showed predominantly weak correlations (|r| < 0.7) (Fig. 3A). GTV *volume* did not differ significantly among the patient groups, which is consistent with the stable mean group trajectories observed during treatment (Fig. 3B). Individual patient trajectories for GTV volume are shown in Fig. S4B.

**Fig. 3:**
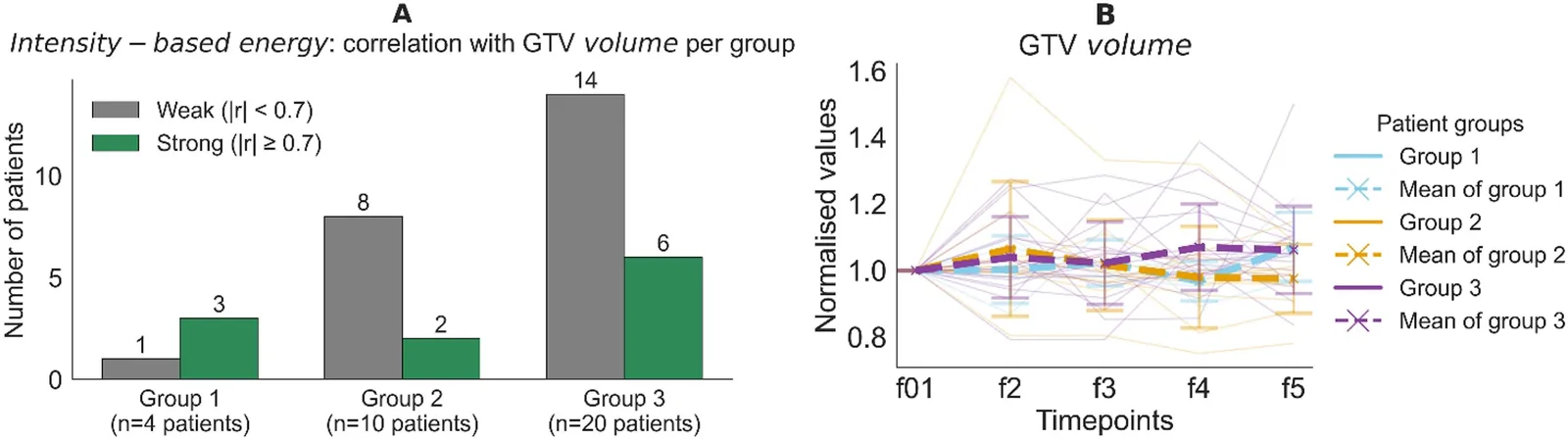
Correlation between gross tumour volume (GTV) *intensity-based energy* and *volume*, and longitudinal evolution of GTV *volume*. (A) Number of patients per patient group showing weak (|r| < 0.7, grey) versus strong (|r| ≥ 0.7, green) correlations between GTV *intensity-based energy* and *volume*. (B) Temporal trajectories of GTV *volume* across the three patient groups (group 1: blue, *n* = 4; group 2: yellow, *n* = 10; group 3: purple, *n* = 20). Solid lines indicate individual patient trajectories, dashed lines represent group means, and error bars denote the standard deviation within each group. (For interpretation of the references to colour in this figure legend, the reader is referred to the web version of this article.)

For the liver ring analysis, we focused on the first (innermost) ring, located 2 cm from the PTV (Fig. S1), which received on average 17.6% of the prescription dose after accounting for the individual fractionation schemes (Fig. S2). From 160 features, after excluding the morphological ones, 138 were initially retained. Following the same pipeline as for the GTV, highly correlated features and features not considered clinically relevant were removed, resulting in 49 features included in the subsequent analysis.

Based on the temporal feature slopes, UMAP (3D projection) followed by HDBSCAN clustering identified two patient groups (Fig. 4): group 1 (*n* = 19) and group 2 (*n* = 15). The corresponding DBCV score was 0.74. For the remaining four outer liver rings (4–10 cm from the PTV), UMAP and HDBSCAN identified between two and three patient groups, with DBCV scores ranging from 0.488 to 0.947 (Fig. S5).

**Fig. 4:**
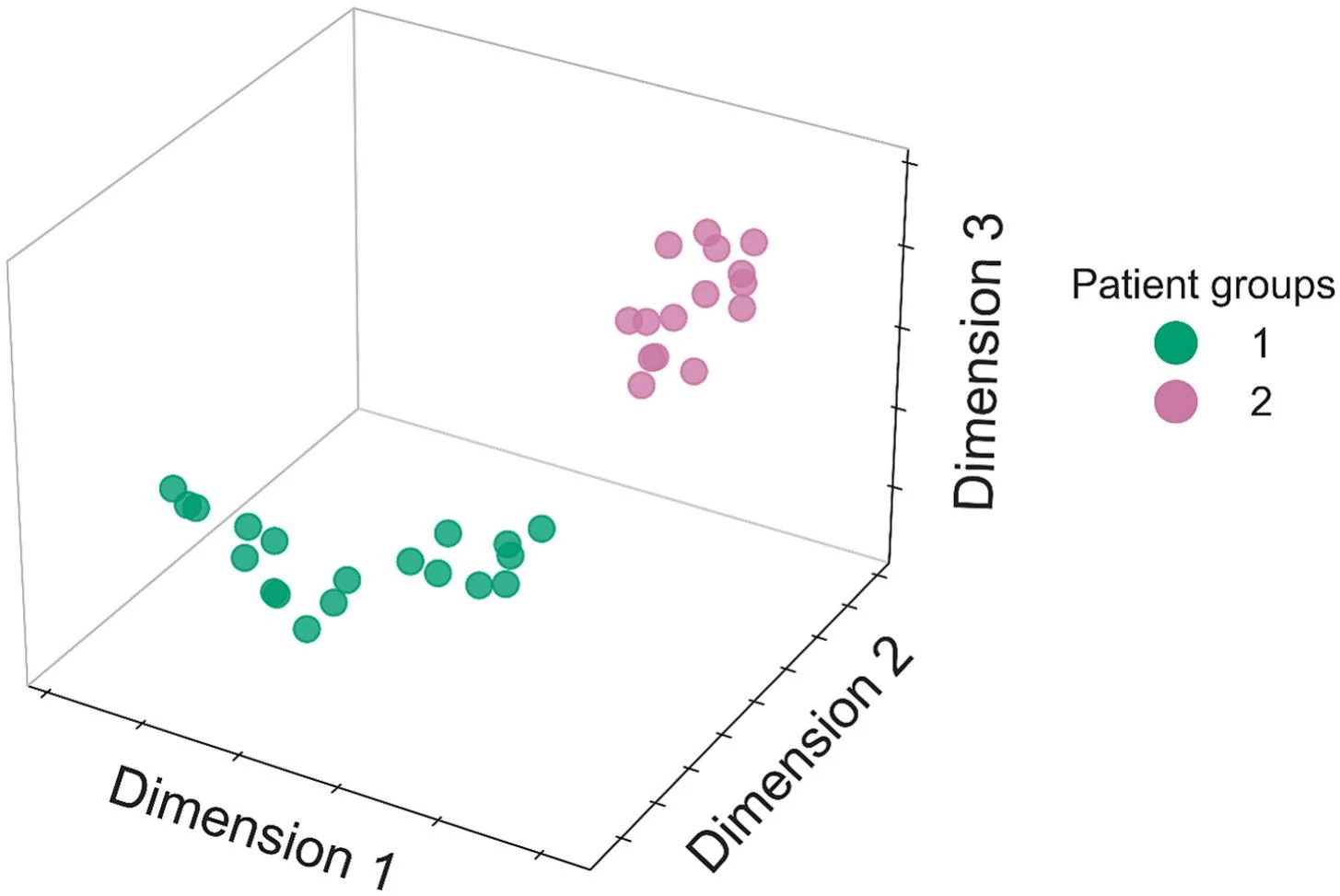
Three-dimensional uniform manifold approximation and projection (UMAP) of patient groups based on the innermost ring features. Hierarchical density-based spatial clustering of applications with noise (HDBSCAN) based on temporal feature slopes for the innermost ring identified two groups: group 1 (green, *n* = 19) and group 2 (pink, *n* = 15). (For interpretation of the references to colour in this figure legend, the reader is referred to the web version of this article.)

Among the evaluated features extracted from the innermost ring, three intensity-based statistical features showed significant differences in their longitudinal evolution between the two patient groups: *intensity-based energy* and *mean intensity* (adjusted *p* < 0.001) and *maximum intensity* (adjusted *p* = 0.005). The corresponding longitudinal group-level trajectories, normalised to the f_01_ values, are shown in Fig. 5, with individual patient trajectories shown in Fig. S7A. The two patient groups demonstrated divergent longitudinal trajectories, with subtle but consistent separation over time that became more visible after the third fraction. Figs. S6-S7 show the corresponding longitudinal patterns for the outer four rings.

**Fig. 5:**
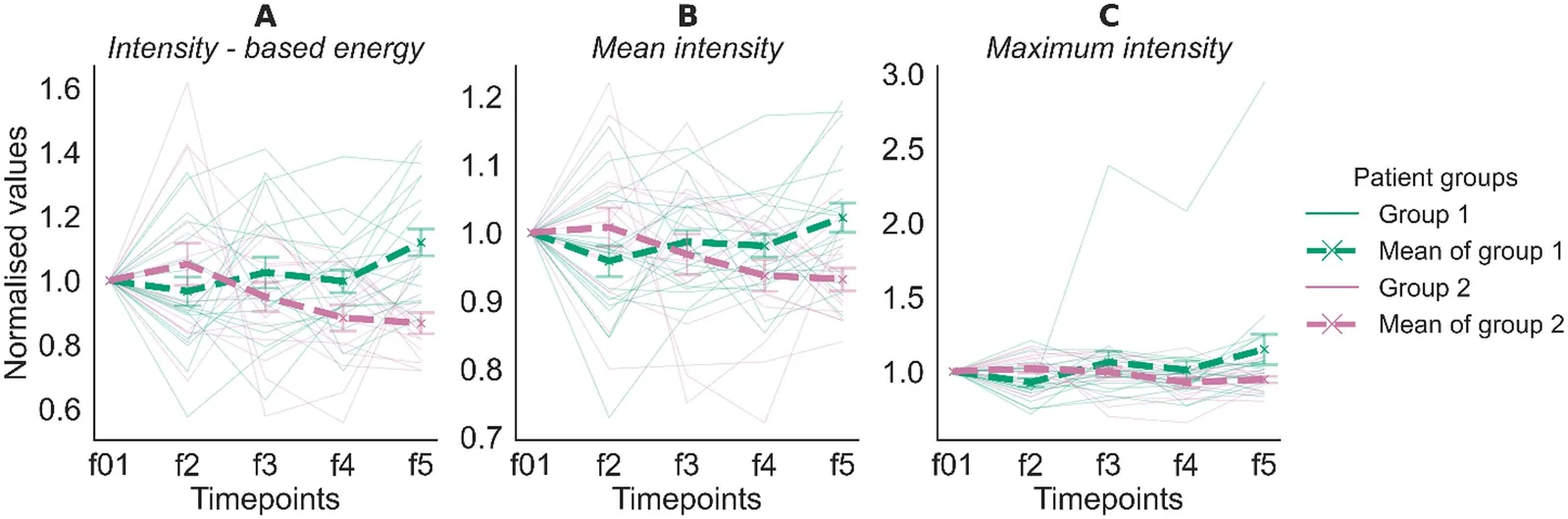
Longitudinal evolution of innermost liver ring features. Temporal trajectories of three representative features from the innermost liver ring are shown across the two patient groups (group 1: green, *n* = 19 and group 2: pink, *n* = 15): *intensity-based energy* (A), *mean intensity* (B) and *maximum intensity* (C). Solid lines indicate individual patient trajectories, dashed lines represent group means, and error bars denote the standard deviation within each group. (For interpretation of the references to colour in this figure legend, the reader is referred to the web version of this article.)

The three features exhibited a higher number of weak correlations with the mean cumulative dose to the innermost ring (|r| < 0.7) in both patient groups, as shown in Fig. 6. Fig. S8 shows the corresponding analyses for the outer liver rings, all of which demonstrated similar patterns of predominantly weak dose–feature correlations.

**Fig. 6:**
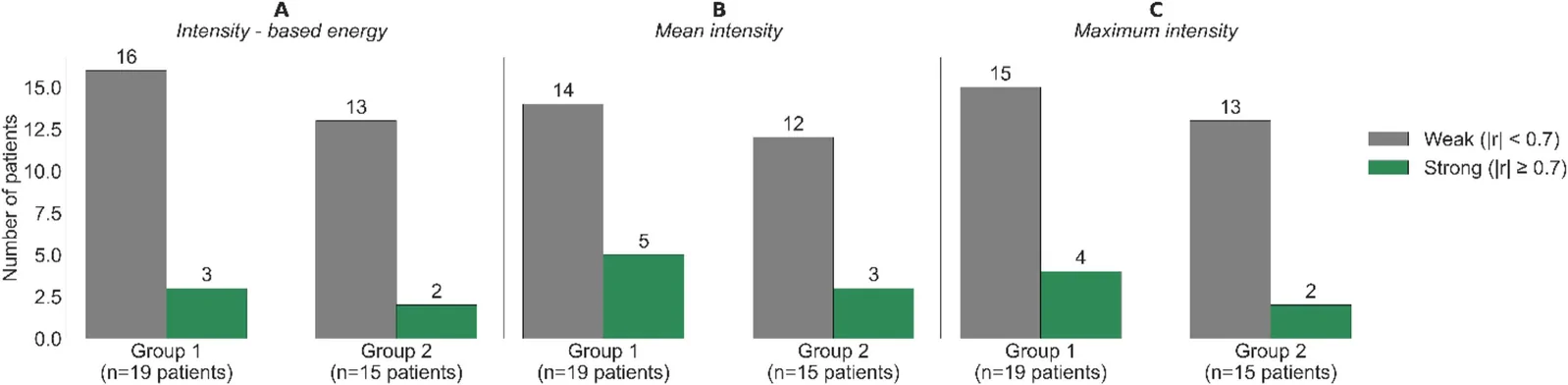
Correlation of innermost liver ring features with the corresponding mean cumulative dose. Bars show the number of patients per group with weak (|r| < 0.7, grey) versus strong (|r| ≥ 0.7, green) correlations between each feature and the corresponding mean cumulative dose: *intensity-based energy* (A), *mean intensity* (B) and *maximum intensity* (C). (For interpretation of the references to colour in this figure legend, the reader is referred to the web version of this article.)

## 4 Discussion

By leveraging MRgRT to acquire MR images before each treatment fraction, we successfully applied a longitudinal radiomics method that identified distinct treatment-related changes in radiomic features extracted from both the GTV and the surrounding healthy liver tissue.

In the GTV analysis, HDBSCAN clustering based on temporal feature slopes identified three patient groups, with a global DBCV score of 0.58. When evaluating the two larger clusters (group 2, *n* = 10, and group 3, *n* = 20) separately, the DBCV score increased to 0.65, indicating improved cluster separation. The smallest cluster (*n* = 4 patients) partially overlapped with the other groups, consistent with the lower global DBCV score and suggesting greater internal variability or noise (Fig. 1). After testing multiple parameter settings, this grouping was found to be the most stable. The resulting imbalance in group sizes reflects the dominant longitudinal patterns captured by the larger patient groups.

Kruskal–Wallis testing revealed that only one GTV feature, *intensity-based energy*, exhibited a statistically significant difference in temporal evolution across the patient groups after FDR correction (adjusted *p* = 0.001, Fig. 2 and Fig. S4A). However, its biological interpretation remains challenging. *Intensity-based energy* is a non-specific imaging feature influenced by both signal distribution and ROI size. The group differences may reflect changes in tissue water content, vascularity, inflammation, cellular composition, or treatment-related cell injury, but these mechanisms cannot be distinguished using the non-quantitative TRUFI sequence and should therefore be presented as hypothesis-generating. Prescription dose could influence treatment-related GTV changes and thereby contribute to longitudinal radiomic trajectories. However, its influence could not be assessed due to the uneven distribution across dose regimens. The strength of the association between GTV *intensity-based energy* and *volume* was group-dependent, with predominantly strong correlations in group 1 and mostly weak correlations in groups 2 and 3 (Fig. 3A). Notably, in the two larger groups, the longitudinal behaviour of *intensity-based energy* is largely independent of macroscopic tumour size changes.

The same longitudinal radiomics pipeline was applied independently to each liver ring. Five concentric 2-cm-wide rings were generated at increasing distances from the PTV to assess spatially resolved treatment effects. This width balanced tissue volume and spatial separation from the target. We focused on the innermost liver ring, 2 cm from the PTV, as it is closest to steep dose gradients and most likely to exhibit radiation-related effects.

Similar to the GTV analysis, *intensity-based energy* emerged as the most discriminative feature between the patient groups (Fig. 5A and Figs. S6–S7) across all liver rings. Two other intensity-based statistical features, *maximum intensity* and *mean intensity*, and one texture feature, *large zone high grey level emphasis*, also showed significant differences between the groups. These features also exhibited weak associations with the mean cumulative dose to the liver rings (Fig. 6 and Fig. S8). This may reflect the relatively low average doses, the use of mean dose across large liver rings, and potentially nonlinear or delayed tissue responses. Technical variability from respiratory position and MRI acquisition, as well as concurrent systemic therapy or pre-existing steatosis/fibrosis, should also be acknowledged and may have influenced the findings in individual patients.

Despite increasing distance from the target volume (Fig. S1), at least one feature in each liver ring exhibited significant longitudinal changes. This suggests that treatment-related changes can be detected even in more peripheral liver tissue, capturing a broader treatment response that is not confined to high-dose regions.

This longitudinal radiomics framework could support several clinical applications. Treatment-related changes in the GTV or surrounding liver tissue warrant further investigation as potential imaging-based indicators of radiation-induced liver injury. Because feature changes in the surrounding liver were only weakly related to dose, monitoring these trajectories may capture treatment response information not explained by dosimetric data alone. In MR-guided adaptive radiotherapy, validated longitudinal radiomics trajectories could ultimately inform adaptive dose modification, for example prompting plan adaptation when healthy liver tissue shows early signs of change.

Our study has limitations. The cohort was relatively small (*n* = 34 patients). While it exceeds that of Jin et al. (*n* = 22) [15], which included primary liver cancers and metastases treated with MR-guided SBRT, our cohort consisted of only liver metastases from a broad range of primary tumour sites (Table S1 and Fig. S3), introducing further biological heterogeneity. The identified patient groups should therefore be considered exploratory, and a larger cohort would improve clustering performance and subgroup analysis. Furthermore, all images were acquired using the 0.35 T TRUFI sequence specific to the ViewRay MRIdian system [5]. Given the differences in field strength, signal-to-noise ratio, and image contrast, our findings may not directly generalise to conventional diagnostic MRI, 1.5 T or 3 T MR-Linac systems. Although preprocessing steps were implemented to reduce and account for baseline variability, the relative nature of MR signal intensities remains a limitation. Validation is therefore required before these changes can guide clinical decision-making.

Due to the absence of clinical outcome data, we were unable to assess how feature changes are associated with outcomes such as PFS or cCR, as previous radiomics studies have done [13], [14], [15], [16], [17], [18], [19]. Although OS data were available, most deaths were attributable to the primary cancer, limiting the relevance of OS as an outcome in this study. In addition, as no toxicities above grade 2 were observed, correlation analyses with clinically relevant toxicity endpoints were not feasible in this cohort.

Compared with previous work [13], [14], [15], this study focused on the longitudinal evolution of features using their temporal slope values, enabling a more detailed characterisation of radiomic changes over time. We also extended our investigation to include the analysis of surrounding liver tissue, providing a more comprehensive view of radiation-induced effects beyond the GTV.

In conclusion, the identification of intensity-based energy as a driving feature in both the GTV and surrounding healthy liver analyses, with significant longitudinal differences between patient groups, suggests its relevance as a candidate imaging biomarker. Given the absence of clinical outcome or toxicity data in this study, however, this remains a hypothesis to be tested rather than a validated conclusion. This aligns with prior findings, such as those by Tomaszewski et al. [14], who also reported an intensity-based statistical feature (*skewness*) as predictive of PFS. Future studies incorporating clinical outcome and toxicity endpoints are needed to determine whether the longitudinal MR-based radiomics framework developed here can be incorporated into models predicting treatment response and radiation-induced toxicity.

## Declaration of generative AI and AI-assisted technologies in the manuscript preparation process

The authors did not use generative AI or AI-assisted technologies in the preparation of this manuscript.

## CRediT authorship contribution statement

**Alina Paunoiu:** Writing – review & editing, Writing – original draft, Visualization, Methodology, Investigation, Formal analysis. **Maksym Fritsak:** Writing – review & editing, Software, Methodology. **Hubert S. Gabryś:** Writing – review & editing, Software, Methodology. **Sebastian M. Christ:** Writing – review & editing, Data curation, Conceptualization. **Matthias Guckenberger:** Writing – review & editing, Funding acquisition. **Stephanie Tanadini-Lang:** Writing – review & editing, Supervision, Project administration, Methodology, Funding acquisition, Conceptualization. **Riccardo Dal Bello:** Writing – review & editing, Supervision, Project administration, Methodology, Data curation, Conceptualization.

## Presentations

Abstracts of this work were accepted as poster presentations at the 28th Annual SASRO Meeting 2024 and ESTRO 2025.

## Ethics declaration

Written informed consent to take part in the study and to publish the article has been obtained from all participants or their legal representatives. The privacy rights of participants have been observed.

This study was performed in compliance with relevant laws, regulatory frameworks and guidelines where the research took place.

This study was conducted in accordance with Declaration of Helsinki.

This study was approved by the Cantonal Ethics Committee Zurich.

(Approval No. BASEC-Nr. 2018-01794)

## Code availability

Not applicable.

## Funding

This work was supported by the Foundation for Applied Cancer Research (Stiftung für angewandte Krebsforschung).

## Declaration of competing interest

The authors declare that they have no known competing financial interests or personal relationships that could have appeared to influence the work reported in this paper.

## References

[1] Fiz F., Viganò L., Gennaro N., Costa G., La Bella L., Boichuk A. (2020). Radiomics of liver metastases: a systematic review. Cancers (Basel).

[2] Mayinger M., Ludwig R., Christ S.M., Dal Bello R., Ryu A., Weitkamp N. (2021). Benefit of replanning in MR-guided online adaptive radiation therapy in the treatment of liver metastasis. Radiat Oncol.

[3] Zhang S., Zhan W., Zeng N., Yang J., Xiong M., Liao W. (2023). Dosimetric comparison in sparing normal tissue dosage by using auto-SBRT planning in oligo liver tumors. Front Oncol.

[4] Jaksic N., Modesto A., Meillan N., Bordron A., Michalet M., Riou O. (2024). Stereotactic body radiation therapy for liver metastases in oligometastatic disease. Cancer Radiother.

[5] Klüter S. (2019). Technical design and concept of a 0.35 T MR-Linac. Clin Transl Radiat Oncol.

[6] Bläker H., Hofmann W.J., Theuer D., Otto H.F. (2001). Pathohistologische Befunde bei Lebermetastasen [Pathohistological findings in liver metastases]. Radiologe.

[7] van Timmeren J.E., Chamberlain M., Krayenbuehl J., Wilke L., Ehrbar S., Bogowicz M. (2020). Treatment plan quality during online adaptive re-planning. Radiat Oncol.

[8] Zwanenburg A., Vallières M., Abdalah M.A., Aerts H.J.W.L., Andrearczyk V., Apte A. (2020). The image biomarker standardization initiative: standardized quantitative radiomics for high-throughput image-based phenotyping. Radiology.

[9] Gillies R.J., Kinahan P.E., Hricak H. (2016). Radiomics: images are more than pictures, they are data. Radiology.

[10] Lambin P., Rios-Velazquez E., Leijenaar R., Carvalho S., van Stiphout R.G., Granton P. (2012). Radiomics: extracting more information from medical images using advanced feature analysis. Eur J Cancer.

[11] Aerts H.J., Velazquez E.R., Leijenaar R.T., Parmar C., Grossmann P., Carvalho S. (2014). Decoding tumour phenotype by noninvasive imaging using a quantitative radiomics approach. Nat Commun.

[12] Yip S.S.F., Liu Y., Parmar C., Li Q., Liu S., Qu F. (2017). Associations between radiologist-defined semantic and automatically computed radiomic features in non-small cell lung cancer. Sci Rep.

[13] Boldrini L., Cusumano D., Chiloiro G., Casà C., Masciocchi C., Lenkowicz J. (2019). Delta radiomics for rectal cancer response prediction with hybrid 0.35 T magnetic resonance-guided radiotherapy (MRgRT): a hypothesis-generating study for an innovative personalized medicine approach. Radiol Med.

[14] Tomaszewski M.R., Latifi K., Boyer E., Palm R.F., El Naqa I., Moros E.G. (2021). Delta radiomics analysis of magnetic resonance guided radiotherapy imaging data can enable treatment response prediction in pancreatic cancer. Radiat Oncol.

[15] Jin W.H., Simpson G.N., Dogan N., Spieler B., Portelance L., Yang F. (2022). MRI-based delta-radiomic features for prediction of local control in liver lesions treated with stereotactic body radiation therapy. Sci Rep.

[16] Sun R., Limkin E.J., Vakalopoulou M., Dercle L., Champiat S., Han S.R. (2018). A radiomics approach to assess tumour-infiltrating CD8 cells and response to anti-PD-1 or anti-PD-L1 immunotherapy: an imaging biomarker, retrospective multicohort study. Lancet Oncol.

[17] Mu W., Tunali I., Gray J.E., Qi J., Schabath M.B., Gillies R.J. (2020). Radiomics of ^18^F-FDG PET/CT images predicts clinical benefit of advanced NSCLC patients to checkpoint blockade immunotherapy. Eur J Nucl Med Mol Imaging.

[18] Vuong D., Bogowicz M., Denzler S., Oliveira C., Foerster R., Amstutz F. (2020). Comparison of robust to standardized CT radiomics models to predict overall survival for non-small cell lung cancer patients. Med Phys.

[19] Bogowicz M., Jochems A., Deist T.M., Tanadini-Lang S., Huang S.H., Chan B. (2020). Privacy-preserving distributed learning of radiomics to predict overall survival and HPV status in head and neck cancer. Sci Rep.

[20] Paunoiu A., Fritsak M., Gabrys H., Guckenberger M., Tanadini-Lang S., Dal Bello R. (2025). Longitudinal analysis of radiomic features in liver cancer patients treated with magnetic resonance-guided radiotherapy (MRgRT). Radiother Oncol.

[21] World Medical Association World Medical Association (2013). Declaration of Helsinki: ethical principles for medical research involving human subjects. JAMA.

[22] Radiomics Team of Radiation Oncology Department at University Hospital Zurich (2025). Z-Rad. Version 25.2 [software]. https://github.com/medical-physics-usz/z-rad.

[23] Fritsak M., Bogowicz M., Vuong D., Dietsche F., Joye A., Guckenberger M. (2025). Z-Rad: the Swiss pocket knife for radiomics. Radiother Oncol.

[24] Yang Y., Sun H., Zhang Y., Zhang T., Gong J., Wei Y. (2021). Dimensionality reduction by UMAP reinforces sample heterogeneity analysis in bulk transcriptomic data. Cell Rep.

[25] Chicco D., Sabino G., Oneto L., Jurman G. (2025). The DBCV index is more informative than DCSI, CDbw, and VIASCKDE indices for unsupervised clustering internal assessment of concave-shaped and density-based clusters. PeerJ Comput Sci.

