# Supplementary material for: Longitudinal radiomics of liver metastases during magnetic resonance-guided radiotherapy
Source: Phys Imaging Radiat Oncol. 2026 Sep 26;41:101094. doi: 10.1016/j.phro.2026.101094 (PMC13651652; doi:10.1016/j.phro.2026.101094)
Supplement: Supplementary file 1 [file mmc1.docx]

### **Supplementary material**

Figure S1. Longitudinal magnetic resonance (MR)-based radiomics workflow. The process includes (1) patient selection (n = 34 patients); (2) MR image acquisition from pre-treatment simulation (f_0_) through five treatment fractions (f_1_) to fraction 5 (f_5_) with gross tumour volume (GTV) and liver ring delineations; (3) image processing (N4 bias field correction (BFC), z-score normalisation and resampling); (4) radiomics feature extraction (shape, intensity and texture features) ([1]); and (5) image analysis. The analysis pipeline examines GTV features (through filtering, patient clustering, longitudinal analysis and correlation with the volume feature), and applies the same processing and clustering pipeline to analyse the liver ring features and their correlation with the corresponding mean cumulative radiation dose.

Table S1. Patient characteristics.

|  | All patients (n = 34) |
| --- | --- |
| **Sex** |  |
| Male | 20 |
| Female | 14 |
| **Age at the time of treatment, median (range)** | 59 (23-81) |
| **Primary tumour** |  |
| Gastrointestinal (GI) | 7 |
| Breast | 5 |
| Lung | 4 |
| Biliary | 3 |
| Head-and-neck (HN) | 3 |
| Gynecological | 4 |
| Other (renal cell carcinoma, neuroendocrine tumours, sarcomas, melanoma, and urothelial carcinoma) | 8 |

Figure S2. Distribution of prescription dose regimens. Bars show the number of patients treated with each fractionation scheme prescribed to the 65% isodose level.

Figure S3. Distribution of primary tumour histology across patient groups. Stacked bars represent the number of patients per histology class (Breast&Gynecological, Gastrointenstinal (GI)&Biliary, Lung&Head-and-Neck (HN), and Other) for the three patient groups identified in the gross tumour volume (GTV) analysis.

Figure S4. Longitudinal evolution of gross tumour volume (GTV) *intensity-based energy* (A) and volume (B). Solid lines indicate individual trajectories shown for the three patient groups (group 1: blue, n = 4; group 2: yellow, n = 10; group 3: purple, n = 20).

Figure S5: Three-dimensional uniform manifold approximation and projection (UMAP) for the outer healthy liver rings. Hierarchical density-based spatial clustering of applications with noise (HDBSCAN) identified patient groups based on temporal feature slopes: (A) Second ring: group 1 (green, n = 6), group 2 (pink, n = 19), and group 3 (orange, n = 9) (global density-based cluster validation (DBCV) metric = 0.517). (B) Third ring: group 1 (green, n = 15) and group 2 (pink, n = 19) (DBCV = 0.787). (C) Fourth ring: group 1 (green, n = 8), group 2 (pink, n = 9), and group 3 (orange, n = 17) (global DBCV = 0.488). (D) Fifth ring: group 1 (green, n = 17) and group 2 (pink, n = 17) (DBCV = 0.947).

Figure S6: Longitudinal evolution of representative features across patient groups for the outer healthy liver rings. (A) Second ring: group 1 (green, n = 6), group 2 (pink, n = 19), and group 3 (orange, n = 9). (B) Third ring: group 1 (green, n = 15) and group 2 (pink, n = 19). (C) Fourth ring: group 1 (green, n = 8), group 2 (pink, n = 9), and group 3 (orange, n = 17). (D) Fifth ring: group 1 (green, n = 17) and group 2 (pink, n = 17). Solid lines indicate individual patient trajectories, dashed lines represent group means, and error bars denote the standard deviation within each group.

Figure S7: Longitudinal evolution of representative features across patient groups for all healthy liver rings. Solid lines indicate individual trajectories shown for each patient group. (A) First ring: group 1 (green, n = 19) and group 2 (pink, n = 15). (B) Second ring: group 1 (green, n = 6), group 2 (pink, n = 19), and group 3 (orange, n = 9). (C) Third ring: group 1 (green, n = 15) and group 2 (pink, n = 19). (D) Fourth ring: group 1 (green, n = 8), group 2 (pink, n = 9), and group 3 (orange, n = 17). (E) Fifth ring: group 1 (green, n = 17) and group 2 (pink, n = 17).

Figure S8: Correlation of outer liver ring features with the corresponding mean cumulative dose. Bars show the number of patients with weak (|r|<0.7, grey) versus strong (|r|≥0.7, green) correlations for representative features across patient groups: (A) second ring, (B) third ring, (C) fourth ring, and (D) fifth ring.

**References**

[1] Figures extracted from the radiomics research group website (<https://www.physik.uzh.ch/en/groups/unkelbach/Research/Radiomics.html>). Accessed April 9, 2026.
